# Scaling up delivery of contraceptive implants in sub-Saharan Africa: operational experiences of Marie Stopes International

**DOI:** 10.9745/GHSP-D-13-00116

**Published:** 2014-02-04

**Authors:** Susan Duvall, Sarah Thurston, Michelle Weinberger, Olivia Nuccio, Nomi Fuchs-Montgomery

**Affiliations:** a Global Health Consultant, Seattle, WA, USA; b Global Health Consultant, New York, NY, USA; c Marie Stopes International, Washington, DC, USA; d Marie Stopes International, London, UK

## Abstract

Contraceptive implants offer promising opportunities for addressing the high and growing unmet need for modern contraceptives in sub-Saharan Africa. Marie Stopes International (MSI) offers implants as one of many family planning options. Between 2008 and 2012, MSI scaled up voluntary access to implants in 15 sub-Saharan African countries, from 80,041 implants in 2008 to 754,329 implants in 2012. This 9-fold increase amounted to more than 1.7 million implants delivered cumulatively over the 5-year period. High levels of client satisfaction were attained alongside service provision scale up by using existing MSI service delivery channels—mobile outreach, social franchising, and clinics—to implement strategies that broadened access for underserved clients and maintained service quality. Use of adaptive and context-specific service delivery models and attention to key operational components, including sufficient numbers of trained providers, strong supply chains, diverse financing mechanisms, and implant removal services, underpinned our service delivery efforts. Accounting for 70% of the implants delivered by MSI in 2012, mobile outreach services through dedicated MSI provider teams played a central role in scale-up efforts, fueled in part by the provision of free or heavily subsidized services. Social franchising also demonstrated promise for future program growth, along with MSI clinics. Continued high growth in implant provision between 2011 and 2012 in all sub-Saharan African countries indicates the region's capacity for further service delivery expansion. Meeting the expected rising demand for implants and ensuring long-term sustainable access to the method, as part of a comprehensive method mix, will require continued use of appropriate service delivery models, effective operations, and ongoing collaboration between the private, public, and nongovernmental sectors. MSI's experience can be instructive for future efforts to ensure contraceptive access and choice in sub-Saharan Africa, especially as the global health community works to achieve its Family Planning 2020 (FP2020) commitments to expand family planning access to 120 million new users.

## Abstract (precis)

Between 2008 and 2012, Marie Stopes International (MSI) provided 1.7 million contraceptive implants in sub-Saharan Africa as part of a comprehensive method mix, primarily through mobile outreach using dedicated MSI providers and also through social franchising and MSI-run clinics. Large-scale access, quality, and informed choice were key elements of MSI's strategy.

## INTRODUCTION

Availability of contraceptive implants in sub-Saharan Africa expands the family planning options from which women of reproductive age can choose to limit or space their children. Currently, nearly 1 in 3 sub-Saharan African women have an unmet need for family planning, the highest proportion (31%) of any region in the world.^1^ Moreover, only 16% of women in sub-Saharan Africa use modern methods of contraception compared with 67% in Latin America and 60% in Asia.^2^ Yet many women want to use contraception. The demand to limit births has risen among married women in a number of countries in East and Southern Africa and is rising more slowly in West and Central Africa.^3^^–^^6^

Implants, a long-acting and reversible contraceptive method (LARC), offer women a viable and highly effective hormonal method for family planning, providing 3 to 5 years of protection against pregnancy (depending on the type of implant used). With a rate of just 1 unintended pregnancy per 2,000 women, implants are more effective than any other reversible method, including the intrauterine device (IUD).^7^ Easily inserted into the arm by a trained health worker, implants are convenient, discreet, and suitable for nearly all women and family planning intentions (delaying, spacing, and limiting childbearing).^7^

Implants are more effective than any other reversible method.

In sub-Saharan Africa, a growing number of women and sexually active adolescents are using family planning, and many are choosing contraceptive implants. While implants account for just 7% of all contraceptive methods used in the region, interest in implants has risen sharply in less than a decade.^8^ For example, between 2004–05 and 2010–11, use of implants rose 17-fold in Ethiopia, 16-fold in Rwanda, 5-fold in Tanzania, and 2.5-fold in Malawi.^7^

A number of factors help explain this dramatic increase:

1. Women's desire to limit family size and growing acceptability of modern methods^6^
2. Wider availability of implants through the introduction of the cost-competitive implant, Sino-implant (II), and the subsequent launch of public-private partnerships,^7^^,^^9^^,^^10^ resulting in price-volume guarantees for Implanon and Jadelle
3. Growing awareness of the benefits of implants among sub-Saharan African women and growing interest in long-acting methods^5^^,^^7^
4. Prioritization of family planning and increasing availability of implants by the donor community and development organizations, including government policy makers^7^^,^^11^^,^^12^

Within this favorable environment, Marie Stopes International (MSI), an international nongovernmental organization (NGO) committed to broadening women's contraceptive choices around the world, has successfully scaled up its delivery of implants in recent years to meet growing demand in sub-Saharan Africa and help clients gain access to information to make informed family planning choices. (We define scale up as an increase in the number of clients using implants, measured by the number of implants delivered.) MSI offers implants as one of many family planning options, including other LARCs, voluntary permanent methods, and short-acting methods. MSI counsels clients on the full range of available methods, so they can choose the method that best fits their lifestyle and family planning goals in accordance with the principles of informed choice and reproductive rights outlined at the Cairo International Conference on Population and Development and underpinning U.S. Government support for voluntary family planning programs.^13^^,^^14^

**Figure:**
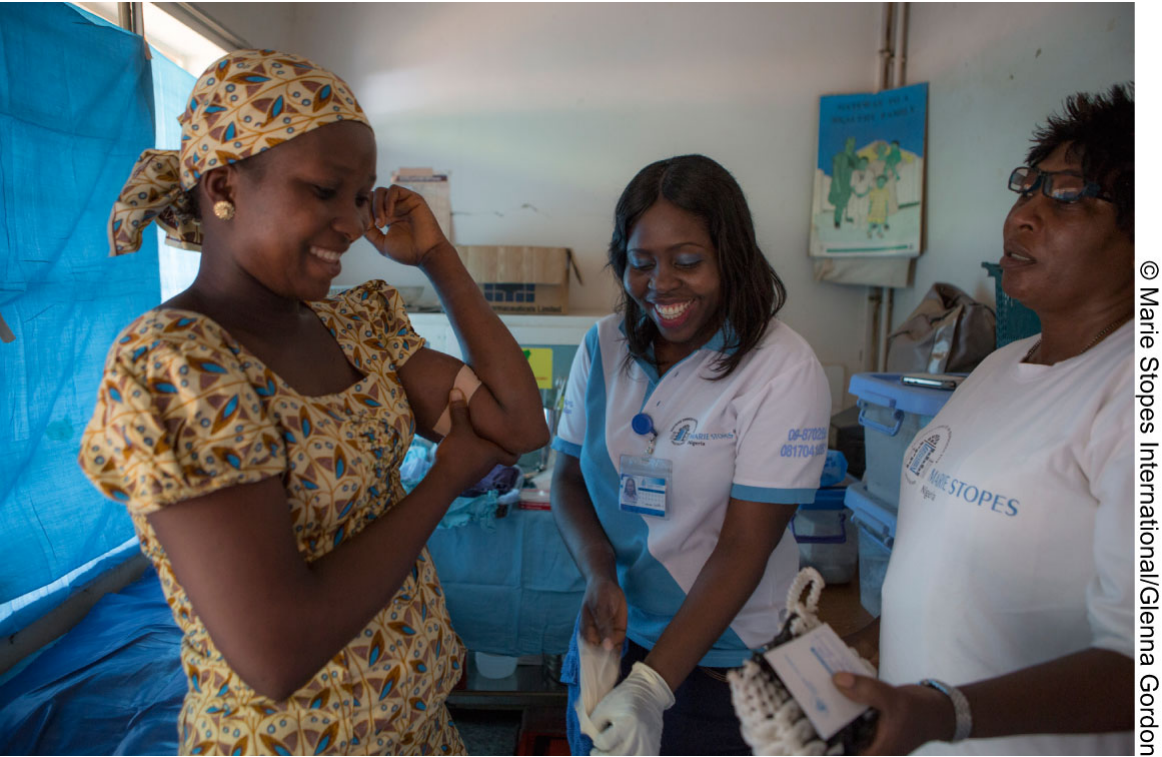
In Nigeria, a family planning client has her contraceptive implant inserted by Marie Stopes International (MSI) providers. Provision of implants by MSI increased more than 10-fold in Nigeria between 2009 and 2012.

## MSI HELPS TO EXPAND ACCESS TO IMPLANTS

In 2008, MSI provided 80,041 implants in the 15 sub-Saharan African countries where we work. In just 5 years, we increased this number considerably to 754,329 implants provided in 2012 (Table 1). Cumulatively, during the 5-year period, MSI delivered more than 1.7 million contraceptive implants in these countries.

Between 2008 and 2012, MSI provided more than 1.7 million contraceptive implants in 15 sub-Saharan African countries.

**TABLE 1.** Number of Implants Provided by MSI in Selected sub-Saharan African Countries,^a^ 2008–2012

**MSI Country Program**	**2008**	**2009**	**2010**	**2011**	**2012**	**% Growth (2011–12)**
Burkina Faso	N/A	2,440	7,835	7,086	14,386	103%
Ethiopia	14,286	31,953	45,737	68,347	88,206	29%
Ghana	2,602	5,549	3,117	14,433	23,162	60%
Kenya	6,652	43,330	69,651	72,477	117,106	62%
Madagascar	6,206	17,535	26,899	34,175	65,229	91%
Malawi	1,719	1,369	2,595	21,691	84,389	289%
Mali	30	3,295	10,588	17,649	33,019	87%
Nigeria	N/A	1,184	5,944	6,388	12,749	100%
Senegal	N/A	N/A	N/A	535	6,600	1,134%
Sierra Leone	N/A	8,387	21,792	29,257	37,672	29%
South Sudan	N/A	N/A	N/A	153	1,138	644%
Tanzania	25,457	28,157	24,465	36,705	64,752	76%
Uganda	13,730	29,875	42,498	81,544	143,762	76%
Zambia	639	3,037	4,724	4,457	9,900	122%
Zimbabwe	8,720	16,166	24,862	40,107	52,259	30%
**TOTAL**	**80,041**	**192,277**	**290,707**	**435,004**	**754,329**	**73%**

Abbreviations: MSI, Marie Stopes International; N/A, not available (because the MSI country program had not yet begun providing implants).

a Data from MSI's service delivery statistics for MSI country programs in sub-Saharan Africa that were active in implant service delivery in 2012. Data from Sudan and Swaziland recorded in 2010 and 2011 are not included because these country programs were closed in 2012. (The 2 countries contribute an additional 864 implants in 2010 and 486 in 2011.)

Rapid expansion occurred in several key East and Southern African countries as well as in West Africa, a region where MSI began intensifying its presence as recently as 2007. Kenya, Madagascar, Malawi, and Uganda scaled up provision of implants considerably from 2008 to 2012, resulting in growth rates near or well over 1,000%, with a 49-fold increase in Malawi and an 18-fold increase in Kenya (Table 1). In Uganda, the number of implant users grew from under 20,000 in 2006 to more than 140,000 in 2011 (Box 1). The high growth rates from 2011 to 2012 in all countries indicate that implant service delivery still has room for further expansion.

The high growth rates in implant provision between 2011 and 2012 in sub-Saharan Africa indicate that implant service delivery has room to expand further.

**BOX 1. Marie Stopes Uganda Scales Up Provision of Implants**

**FIGURE 1.**
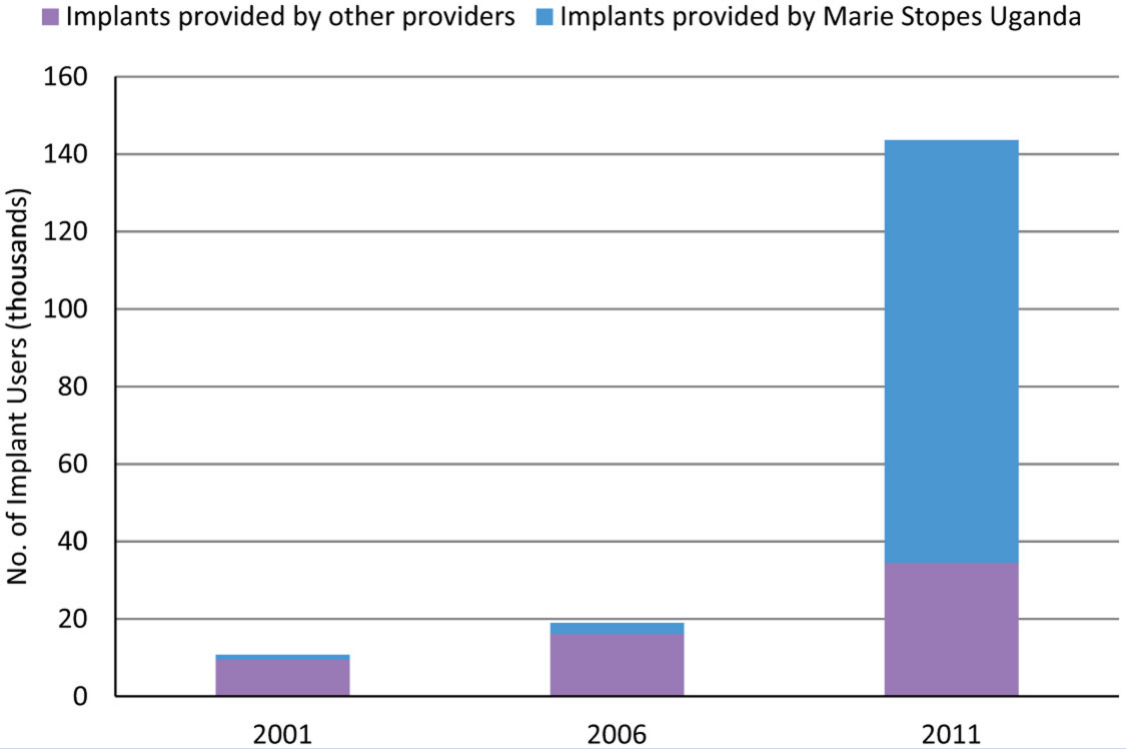
Number of Women Using an Implant Provided by Marie Stopes Uganda Versus Other Providers,^a^ 2001, 2006, and 2011 ^a^ “Other providers” includes all private-sector organizations offering implants, other than Marie Stopes Uganda, and all public-sector providers, including Ministry of Health facilities. Data for Marie Stopes Uganda users are from Marie Stopes International (MSI) service statistics and are modeled using MSI's Impact 2 model. These estimated user numbers include women who received an implant supplied by MSI that year as well as women who received implant services from MSI in past years who are modeled to still be protected by the implant. Data for implants provided by other providers are from 2001, 2006, and 2011 Uganda Demographic and Health Surveys and 2010 UN Population Prospects.

Between 2006 and 2011, Marie Stopes Uganda scaled up provision of implants and, in so doing, increased the size of the overall market for implants in the country. In 2001 and 2006, the total number of implant users in Uganda—comprised of new users and those who had their implants inserted in years prior—remained under 20,000 (Figure 1). Between 2006 and 2011, the number of users expanded more than 7-fold to more than 140,000 users.

By 2011, Marie Stopes Uganda had become the dominant implant provider in the country. We estimate that approximately 3 of every 4 women using an implant in Uganda in 2011 received their method from MSI. When we consider that the number of women choosing family planning in the general population increased by 60% between 2006 and 2011 and that the proportion choosing implants also expanded greatly (from 1 in 50 to 1 in 10), the role of Marie Stopes Uganda in reaching 76% of these users is significant.^8^ These data suggest that our scale-up efforts in implant services likely changed Uganda's national pattern of contraceptive use by 2011.

A number of factors contributed to the growth in implant provision by Marie Stopes Uganda:

1. Strong mobilization of donor resources, including bilateral funding from the U.S. Agency for International Development (USAID)
2. A large expansion in the number of service delivery sites
3. An increase in the number of community campaigns to generate demand for the contraceptive options available from Marie Stopes Uganda, including implants

**FIGURE 2.**
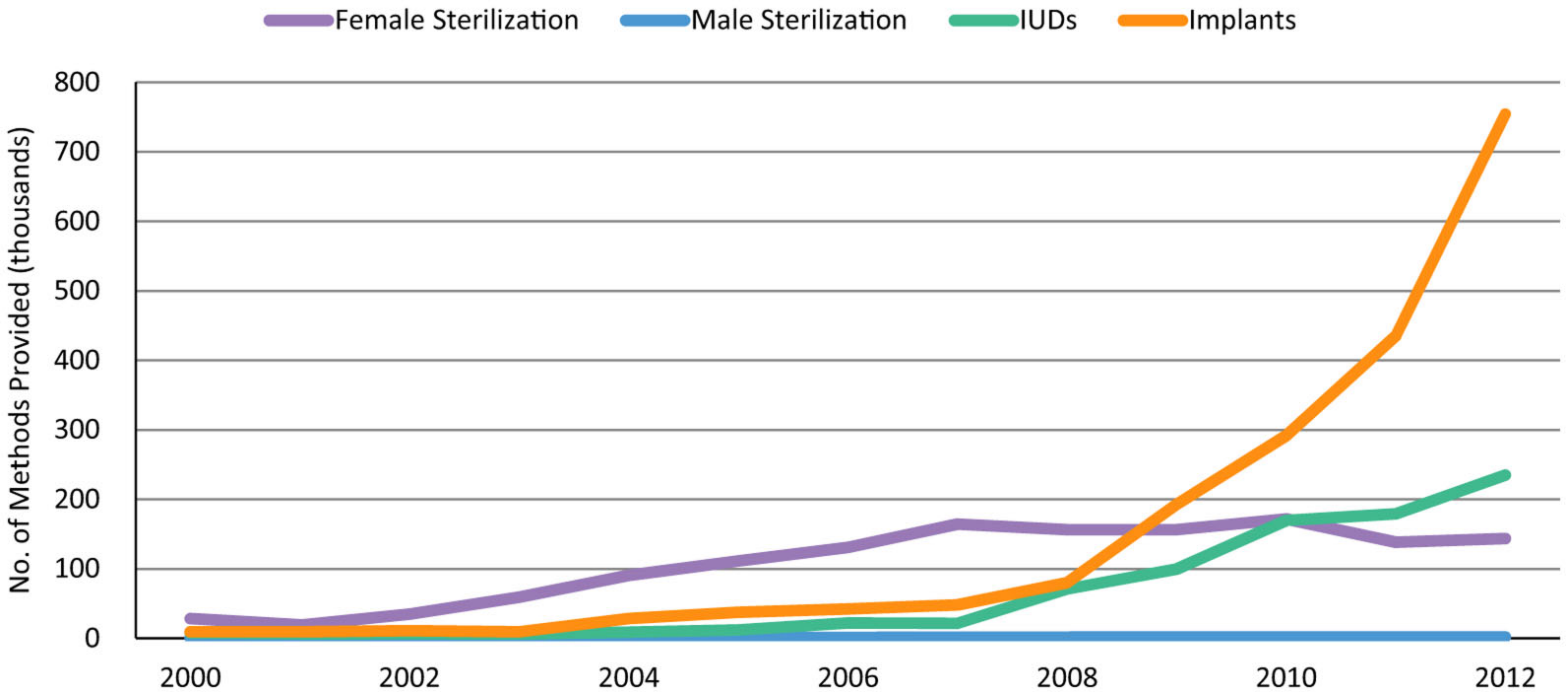
Number of LAPMs Provided by MSI in sub-Saharan Africa, by Method, 2000–2012 Abbreviations: LAPMs, long-acting and permanent methods; MSI, Marie Stopes International. Data from MSI service statistics.

**FIGURE 3.**
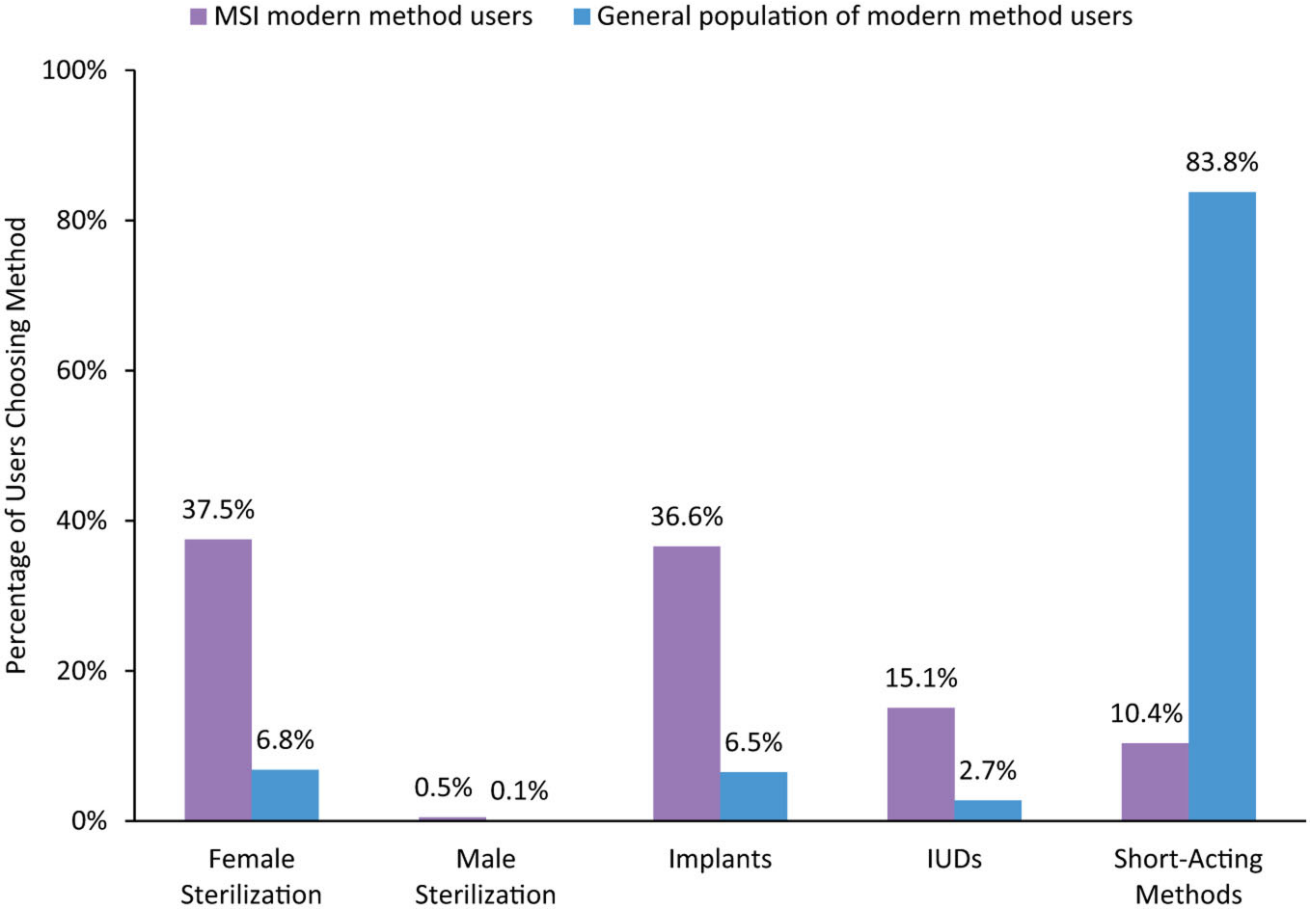
Method Mix Among Modern Method Users, Marie Stopes International (MSI) Users Versus the General Population, in African Countries Where MSI Operates, 2012 Data for MSI users are from MSI service statistics, with user numbers modeled using MSI's Impact 2 model. As explained in the footnote to Figure 1, LAPM users include those who received their method in prior years who continue to be protected. Because sterilization protects women for a longer duration than IUDs and implants, previous sterilization clients remain in the total “user” number for more years (until aging out at 49, based on median age of sterilization). Data for the general population are from Demographic and Health Surveys for those sub-Saharan African countries where MSI operates.^8^ For MSI user numbers, short-acting methods exclude condoms to avoid the risk of overestimating condom use because of user wastage and dual protection.

The steep increase in implant provision between 2008 and 2012 (more than 9-fold) demonstrates a marked difference from our provision of other long-acting and permanent methods (LAPMs) during the same period (Figure 2). Like implants, use of IUDs has steadily increased in sub-Saharan Africa since 2008 due to MSI's overall family planning program scale up in the region. However, stronger demand for implants resulted in a much faster pace of growth in comparison with IUDs. For tubal ligations, the number of services provided per year remained fairly steady over the 5 years. The number of female sterilization users, however, still accounts for the highest proportion of MSI family planning users in the region (Figure 3), because MSI has delivered more tubal ligations than other LAPMs historically; therefore, the estimated number of sterilization users in 2012 reflects these past trends.

MSI's capacity to deliver implant services—and to scale up efforts in response to client demand—complements the existing method mix provided by the public sector and other private-sector providers, helping to meet the needs of clients who prefer implants. Public-sector facilities in sub-Saharan Africa often face constraints in providing LARCs, including implants, on a reliable basis. A lack of adequate infrastructure, frequent commodity stockouts, and a lack of skilled providers hinder public-sector provision.^15^^,^^16^ Moreover, many public- and private-sector family planning programs deliver predominately short-acting methods, and, commercial pharmacies, social marketing programs, and public facilities often offer better access to short-acting methods than to long-acting methods, including implants.

As a result, the method mix of women in the region using an MSI-provided method differs considerably from the method mix of the wider sub-Saharan African population as a whole. In 2012, whereas 83.8% of women of reproductive age in sub-Saharan Africa overall were using a short-acting method, only 10.4% of MSI users were.^8^ In contrast, a far greater proportion of MSI users (36.6%) than the general population (6.5%) were using implants and other LAPMs for their family planning needs (Figure 3).

## MSI SERVICE DELIVERY CHANNELS

MSI has successfully delivered family planning services through a number of channels, including the 3 main channels of:

1. Mobile outreach
2. Social franchising
3. Static clinics

Using more than one service delivery channel broadens the access points for a client, thereby increasing the likelihood that information about family planning choices will reach her and that she will have access to choose a method she wishes.^1^^,^^6^

**FIGURE 4.**
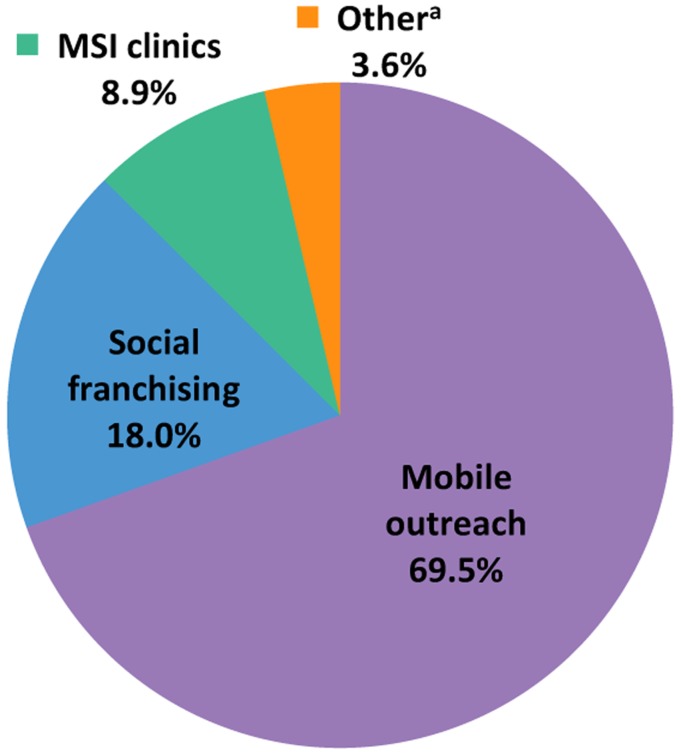
Proportion of Implants Delivered by MSI in sub-Saharan Africa, by Service Delivery Channel, 2012 Abbreviations: MSI, Marie Stopes International. ^a^ “Other” includes community-based distribution, community health workers, and miscellaneous providers. Data from MSI service statistics. Data do not include 1,898 implants delivered through social marketing in Mali.

In 2012, the largest proportion of MSI's implant provision in sub-Saharan Africa was through mobile outreach services (Figure 4). Accounting for nearly 70% of all implants delivered, our outreach services provided almost 4 times as many implants as our social franchisees (18.0%) and nearly 8 times as many as our static clinics (8.9%). Still, the social franchising proportion is notable, since half of our social franchising programs in sub-Saharan Africa were recently established in the latter half of 2012. These results underscore the importance of mobile outreach and social franchising for expanding access to implants as part of a comprehensive method mix.

70% of MSI's implant clients in sub-Saharan Africa were reached through mobile outreach. Social franchising also showed promise, accounting for 18% of implant clients.

Typically, variations or service delivery innovations build on 1 of these 3 channels. The scale of each of these channels also varies by country, depending on client needs and infrastructure availability. Table 2 contains a summary of our country program operations in those sub-Saharan African countries active in implant service delivery in 2012.

**TABLE 2.** Summary of MSI Country Programs Active in Implant Service Delivery in sub-Saharan Africa, 2012

**MSI Country Program**	**Month/Year Program Opened**	**No. of FP Clients (all channels)**	**No. of Implants Provided**	**No. of Mobile Outreach Teams**	**No. of Clinics**	**No. of Social Franchisees**	**Month/Year Social Franchising Started**
Burkina Faso	07/2009	24,517	14,386	4	1	N/A	N/A
Ethiopia	09/1990	206,723	88,206	10	31	443	10/2008
Ghana	10/2006	39,798	23,162	6	5	106	03/2008
Kenya	03/1986	229,836	117,106	15	25	279	04/2004
Madagascar	06/1992	147,661	65,229	46	14	127	11/2009
Malawi	09/1987	229,310	84,389	39	31	54	06/2008
Mali	11/2008	45,787	33,019	7	3	34	06/2012
Nigeria	04/2009	16,446	12,749	5	1	51	09/2012
Senegal	11/2011	9,989	6,600	3	1	10	10/2012
Sierra Leone	03/1988	127,148	37,672	13	12	100	12/2008
South Sudan	08/2011	1,778	1,138	2	2	N/A	N/A
Tanzania	09/1990	149,252	64,752	26	12	N/A	N/A
Uganda	07/1993	260,466	143,762	24	15	419	06/2012
Zambia	06/2008	18,261	9,900	7	3	7	07/2012
Zimbabwe	04/1988	146,680	52,259	9	9	61	08/2012

Abbreviations: FP, family planning; MSI, Marie Stopes International; N/A, not applicable.

Data from MSI service statistics. Number of FP clients were estimated from MSI service statistics, in which each service for a long-acting and permanent method is equal to 1 client and each year's supply of short-acting methods is equal to 1 client.

When determining which channels to use, MSI considers the efficiency and reach of each one within the specific country context. Monitoring both efficiency and reach are essential considerations for enabling service delivery scale up and ensuring scale up is equitable.^1^ **Efficiency** refers to allocating time, effort, and resources strategically in service delivery to maximize the greatest program impact.^17^ Matching the size of a clinic or provider team to client demand and service patterns of a facility or catchment area is one example of efficiency. To measure efficiency, MSI teams use cost per couple-year of protection (CYP), a metric that shows the average cost of delivering a contraceptive method relative to the number of years the method protects against pregnancy. Currently, MSI uses cost per CYP for internal program monitoring and decision making; costing data will be made available in future studies focused on service delivery and scale-up costs. It is important to note that this metric is not simply about minimizing the cost per CYP, but rather about ensuring we use our resources to achieve the most impact—accounting for our role in expanding access and choice, improving quality, and ensuring equity.

**Reach** refers to expanding access to family planning services, meaning that every potential client can obtain services regardless of financial, geographical, and/or cultural barriers.^17^ We select service delivery channels that will reach clients affected by gaps in service outlets or contraceptive methods. At the same time, we consider channels that will enable existing clients to continue and/or switch their methods, if they choose. MSI monitors a program's reach through indicators such as the number of CYPs generated or the number of service delivery sites established. Recently, MSI also began monitoring the number of high-impact CYPs generated by different service delivery channels. Developed by MSI, this indicator measures a program's ability to deliver services to those facing the highest barriers to access, such as the poor, young women, those who have not previously been using family planning (called “adopters”), and users of short-acting methods who seek services at MSI to meet their desire for a LAPM (called “switchers”).

### Mobile Outreach

MSI's mobile outreach services deliver implants and other contraceptive methods through a team of MSI dedicated providers that brings equipment and commodities directly to clients. The use of these dedicated providers—those who fill a specific service delivery gap by focusing primarily on the provision of certain contraceptive methods, such as LAPMs—is a key component of MSI′s mobile outreach strategy.^18^ Unlike some dedicated provider models, we employ MSI staff, not external providers. These teams visit outreach sites on a regular basis, ranging from every 4 to 6 weeks to once per quarter in the most remote regions, expanding access to contraceptive choice through provision of LAPMs during these visits. (In support of informed choice, our dedicated providers refer clients who want short-acting methods to their public-sector counterparts located at the same site when available, or they furnish these methods directly in cases of stockouts at the public facility.)

To help achieve equity, MSI provides underserved clients who do not otherwise have access to implants or other LAPMs with free or highly subsidized family planning services. As a result, the mobile outreach channel often generates high demand and commonly attracts new family planning adopters, a key metric for monitoring scale-up efforts.^19^^–^^21^

In 2012, 41% of mobile outreach family planning clients were adopters, reached through our 216 mobile outreach teams in the sub-Saharan African countries offering implants (Table 2). Moreover, and importantly for implant scale up, 39% of our outreach clients switched from short-acting methods to LAPMs, indicating client preference for longer-acting contraception.^8^

41% of MSI's mobile outreach family planning clients in 2012 were adopters and 39% switched from short-acting methods to long-acting and permanent methods.

Mobile outreach services can also be an effective channel for program scale up in terms of efficiency. By strategically using existing community infrastructure, small teams, and outreach schedules that coincide with client demand, mobile teams can maximize impact from its program inputs. For areas that are not too rural but still hard to reach, this channel has proved to be cost-effective.^22^ Teams of dedicated providers also have been shown to increase the number of IUD and implant insertions, and therefore, program scale up.^18^

Depending on the geography of a particular catchment area, MSI uses either a mobile clinical service team or a mobile community outreach worker team, its 2 primary outreach models.^23^ The **mobile clinical service team** model deploys small teams, typically 3 MSI dedicated providers and a driver, to rural areas for delivery of family planning services in existing health centers (usually public facilities) where possible. Through a collaborative process with local governments, MSI chooses these clinics because of their infrastructure, their ties to the community, and their visibility among clients. Some women also prefer to access family planning at a health center in order to disguise the reason for their visit. If needed, a team uses other community facilities (for example, schools and community centers), or sets up a low-cost, temporary structure such as a tent.

In an effort to serve densely populated urban and peri-urban areas, our second model, the **mobile community outreach worker team**, is a flexible, low-cost adaptation of the clinical service team model. In the community outreach worker team model, a smaller team—often consisting of just 1 or 2 MSI dedicated providers of lower-level cadres—provides implants and other contraceptive methods, often in client homes or other non-health facility locations. A typical example is when 1 paramedic or nurse and 1 family planning counselor will use local transport, rather than MSI-owned vehicles, to reach clients (Box 2). Although the teams for both models are based out of an MSI clinic, they mobilize interest in their services in advance of their arrival in the community through a variety of demand-generation activities (Table 3).

**BOX 2. Marie Stopes Tanzania Develops Innovative Urban Outreach Model**

Throughout its 30-plus year history, Marie Stopes Tanzania reached middle-income urban clients through MSI clinics and low-income rural clients through mobile outreach. However, by 2010, we had identified a growing gap in contraceptive-seeking behavior: our static clinics were not adequately reaching many low-income urban and peri-urban women wishing to use injectables and LARCs, including implants.

MSI's existing rural outreach model consisted of MSI-owned 4x4 vehicles and large clinical teams. Such a model would be too cumbersome in a peri-urban context, and so Marie Stopes Tanzania set out to innovate urban outreach. In 2010, we launched a pilot *bajaji* (motorized auto-rickshaws) outreach model in Zanzibar, with support from USAID. This new urban outreach model, using a team consisting of 1 MSI nurse and 1 *bajaji* driver, is a streamlined and more flexible version of MSI's rural outreach model.

The *bajaji* outreach model significantly reduces startup and operational costs due to lower staffing, fuel, and vehicle expenses. *Bajaji* nurses deliver contraceptive methods directly in clients' homes, in addition to providing family planning services at standard mobile outreach model sites (public health facilities or other community-based static sites). Clients report that these home-based services allow them to circumvent key access challenges, including lack of time to attend clinics, need for discretion in seeking family planning, and, in some contexts, cultural norms requiring women to be accompanied when traveling outside the home.

Within several months of starting *bajaji* services in Zanzibar, the Ministry of Health in Mwanza City invited Marie Stopes Tanzania to expand the model for its underserved urban neighborhoods.

In the 12-month pilot period in Zanzibar, *bajaji* teams delivered family planning services to 3,650 clients, of which 2,122 chose implants. In the 7-month pilot in Mwanza City, *bajaji* teams delivered family planning services to 2,531 clients, of which 1,432 chose implants. Client interest in voluntary permanent methods resulted in 86 referrals to MSI clinics for tubal ligations (73 in Zanzibar and 13 in Mwanza City).

Several MSI country programs in Africa and Asia are currently replicating this model to reach underserved urban and peri-urban clients.

**TABLE 3.** Demand-Generation Activities to Educate Clients About Family Planning and MSI Services, by Channel

**Mobile Outreach Services**	**Social Franchising**	**Clinics**
Delivery of high-quality services to enable word-of-mouth referrals	Delivery of high-quality services to enable word-of-mouth referrals	Delivery of high-quality services to enable word-of-mouth referrals
Educational outreach by community health workers (CHWs) or other community agents about importance of family planning and different methods through: Door-to-door mobilization Group information sessions Educational/promotional communication and media	Educational outreach about family planning and long-acting and reversible contraceptives (LARCs), including implants, as well as about *BlueStar* family planning services through: CHWs and other community agents Print or radio advertisements	Educational outreach about family planning and MSI services through: Kiosks at regular markets and popular events Radio show appearances by MSI clinic staff Flyers and promotional materials available at locations frequented by young women, such as markets, universities, and beauty salons
Designated day for team visit, making it a noteworthy and anticipated community event	Special discount days on LARC services	
Local media advertisements about voluntary family planning and LARCs, including implants	Promotion of *BlueStar* brand, as an overall sign of quality service delivery	Training for all clinic staff including receptionists and support staff to ensure client-friendly, non-judgmental environment
Where appropriate, referrals from other MSI service delivery channels	Referrals from: Other non-MSI services at franchisee Other MSI service delivery channels, where appropriate	Where appropriate, referrals from other MSI service delivery channels
Announcement of upcoming mobile team visit via: Town crier Radio CHWs or other community agents		

Both of these outreach program models are examples of how MSI collaborates with the public sector, building the clinical competencies of public-sector providers and creating synergies between public and private systems. For example, we prepare public providers for assessing and handling any complications that may arise from implant insertions. Such training is critical to meet follow-up needs of clients between visits from the MSI team. To ensure clients receive high-quality follow-up care, MSI coordinates referral networks with higher-level facilities to manage side effects that infrequently arise and that are beyond the capacity of lower-level public-sector providers. In the event that a client experiences a severe side effect, defined as a frequent level of discomfort requiring medical attention, we provide technical expertise and pay for transport and hospital fees if higher-level facility referral is needed. Where possible, we also build the clinical skills of public-sector providers in other ways, focusing on specific areas that need reinforcement (such as client counseling techniques and implant removal protocols).

### Social Franchising

MSI's *BlueStar* social franchise networks* engage existing private providers to deliver high-quality sexual and reproductive health services, including implants, in underserved areas. Contracted to MSI but operated and owned by private providers, these networks are organized under commercial franchising principles, which have been shown to facilitate standardization and increase client volume, including for family planning services.^24^^–^^26^ MSI has adopted a “partial franchising” model for our social franchise networks. In this model, we regulate and support only some of the franchisees' services and commodities, namely the reproductive health and family planning services; the franchisee may offer additional services that we do not oversee.

In sub-Saharan Africa, franchisees are typically located in urban and peri-urban areas as well as towns and trading centers in rural areas. By engaging these existing providers, we leverage and strengthen the health infrastructure and aim to achieve greater health system integration between the public and private sectors. MSI gains access to an established clinic and existing client base in a community when we invite new members to the *BlueStar* network, obviating the need for the startup costs and effort associated with opening a new MSI clinic. At the same time, we expand client access to key services that these private clinics would otherwise not be able to provide adequately, allowing health systems to make better use of the capacity in the private sector to achieve public health-sector goals, such as increases in contraceptive prevalence.

At the individual level, *BlueStar* franchisees increase options for existing contraceptive users as well as increase the market for family planning users and attract new users. In 2012, 78% of our *BlueStar* LAPM clients in sub-Saharan Africa chose implants—135,144 implant clients in 12 countries. Due to this demonstrated potential, social franchise networks will be key channels for scaling up implant services in many MSI country programs in the coming years.

In 2012, 78% of MSI's social franchising LAPM clients chose implants.

To help family planning program scale up and to offer services at affordable prices to our clients at our 1,691 *BlueStar* clinics in sub-Saharan Africa, MSI facilitates access to high-quality implants (and other commodities for other franchised services delivered) in 2 ways. We either supply these implants at a reduced price or negotiate access to pooled commodities at the national level on behalf of franchisees. Discounts vary from country to country. For example, while an MSI subsidy enables our Ghana franchisees to receive implant commodities at the same price as their public-sector counterparts, we are able to supply our Madagascar franchisees with implants (and other contraceptive methods) free of charge.

Prior to joining the *BlueStar* network, individual clinics are not usually in a position to offer implants or other LAPMs to their clients; in most countries, there is no private-sector supply chain for implants outside of social franchise networks. By joining *BlueStar*, the benefits of supply-chain support—namely, more reliable and affordable access to consumables and implants themselves—enable *BlueStar* clinics to provide a wider range of contraceptive methods. These economies of scale result in cost savings for our clients, thereby increasing access for lower-income clients and scaling up equitable service provision.

### MSI Clinics

Clinics have been our longest-standing service delivery channel. Owned and operated by MSI, our clinics are located in cities, towns, and peri-urban areas throughout 42 countries worldwide, with 165 delivering reproductive health and family planning services in the 15 sub-Saharan African countries that provided implants in 2012 (Table 2).

In many of these countries, our clinic services augment the contraceptive method mix available from the public and private sector in urban and peri-urban areas, attracting new clients because of the different services that MSI offers, including implants. In fact, in 2012, 38% of our clinic clients in sub-Saharan Africa were family planning adopters.^8^ Therefore, these clinics are important for expanding implant access to women in their respective catchment areas.

In 2012, 38% of MSI's clinic clients in sub-Saharan Africa were family planning adopters.

MSI clinics offer some advantages to scaling up access to implants over other service delivery channels in terms of efficiency and reach. Because the clinics are well-established in their catchment areas, with appropriate equipment and trained providers, our clinics can offer implant services in a manner that uses program inputs strategically to maximize impact. For example, we can scale up implant service delivery without significantly increasing overhead costs, such as transport with mobile outreach services. In terms of reach, these clinics tend to serve a population that is relatively wealthier than those served by our mobile outreach channel; in 2012, approximately 17% of our clinic clients in sub-Saharan Africa lived on less than US$1.25 per day compared with 42% of our mobile outreach clients.^8^ At the same time, income generated from the sliding scale fees charged by our clinics helps subsidize our outreach service delivery, in which fees are typically not charged.

## QUALITY ASSURANCE MEASURES

All MSI delivery channels prioritize service quality when providing clients with contraceptive methods. High-quality programs yield high levels of client satisfaction, a principal determinant of a client's initial and continued use of family planning services.^27^^–^^30^ The quality level of family planning service delivery, including implant provision, also directly influences the demand generation facilitated by client experiences and word-of-mouth communication, and, in turn, program scale-up efforts.

MSI implements various quality-control activities, such as competency-based training and refresher courses, to train providers on MSI standards. We also train facility staff and outreach teams on how to use MSI's management information system to record client visits, services provided, expenditures, and stock of commodities and equipment. We then use various tools, such as mystery clients, supportive supervision, and audits, to monitor and ensure these service standards are met. See the Appendix for a complete list of MSI's quality-assurance activities. Through these measures, our staff and partners pay attention to quality throughout each stage of service delivery.

As a result of this rigorous attention to quality, MSI clients have reported high rates of satisfaction with the services received, regardless of the channel from which they obtained family planning services. In 2012, MSI family planning clients across 11 sub-Saharan African countries gave our services an average rating of 4.4 on a 5-point Likert scale, in which 5.0 signified “very good.” The highest-rated aspect of service delivery was “friendliness and respect from the health care provider,” followed by “friendliness and respect from staff.” These data are potentially subject to “courtesy bias,” in which the clients are reluctant to express negative opinions to the interviewer.

**FIGURE 5.**
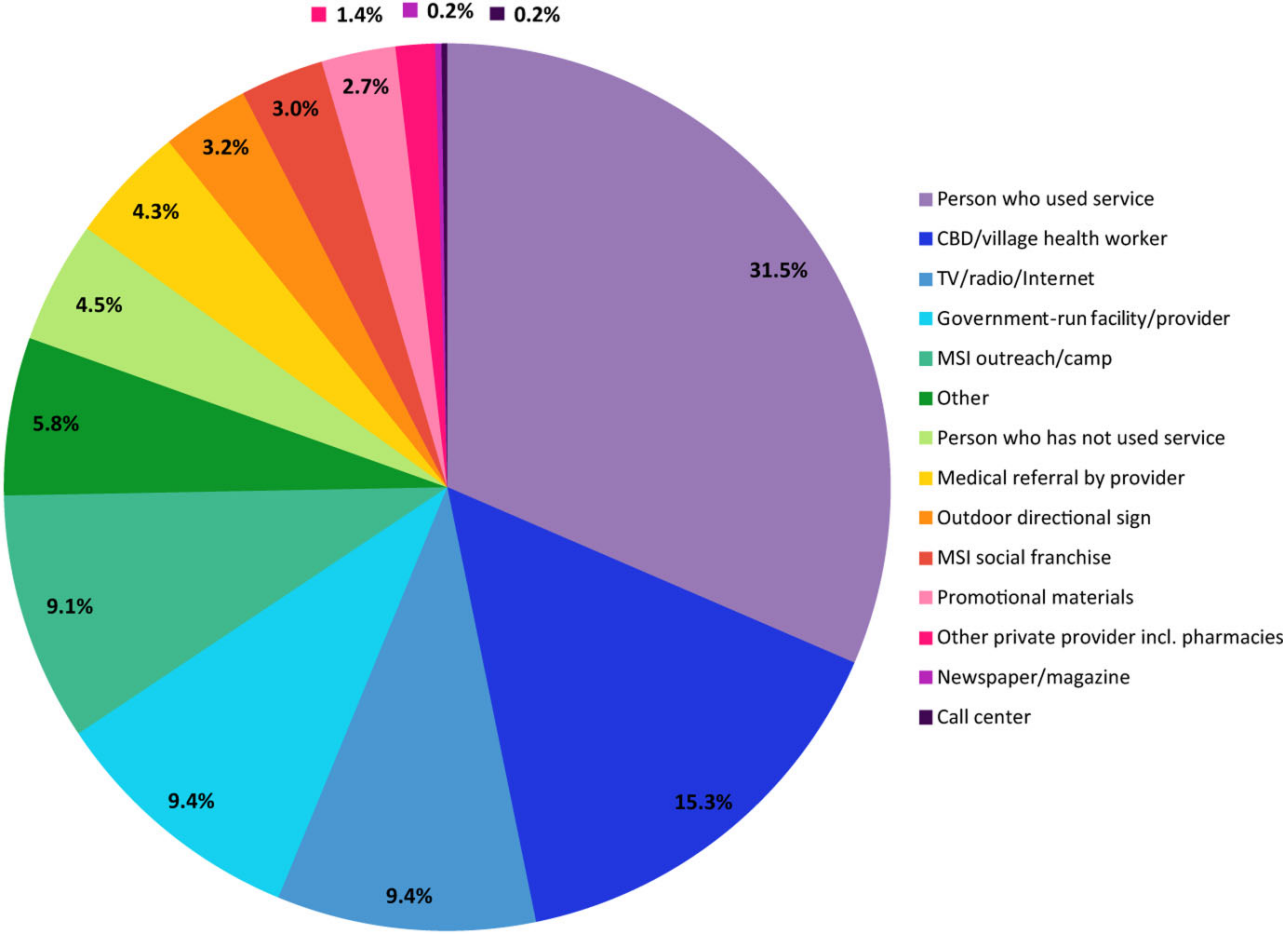
Most Influential Source of Information Affecting Decision to Choose MSI Services Among sub-Saharan African Clients^a^ Across All Service Delivery Channels,^b^ 2012 (N = 6,225) Abbreviations: CBD, community-based distribution; MSI, Marie Stopes International. ^a^ Data from exit interviews in 11 sub-Saharan African countries, from August 2012 through December 2012. ^b^ Results were weighted by region and delivery channel where appropriate. When weighting by delivery channel, data were only used from countries where the relevant delivery channel had been surveyed.

**FIGURE 6.**
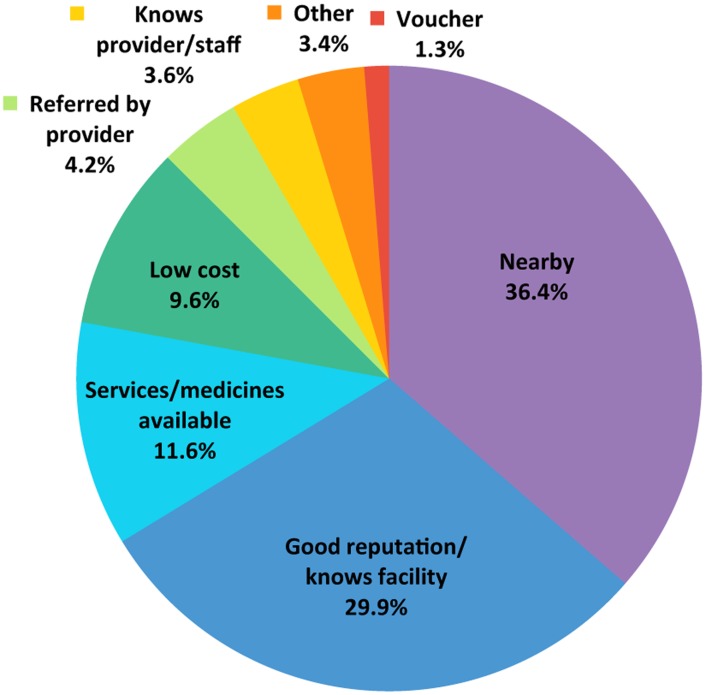
Most Important Reason for Choosing Services From Marie Stopes International Among sub-Saharan African Clients^a^ Across All Service Delivery Channels,^b^ 2012 (N =  6,225) ^a^ Data from exit interviews in 11 sub-Saharan African countries, from August 2012 through December 2012. ^b^ Results were weighted by region and delivery channel where appropriate. When weighting by delivery channel, data were only used from countries where the relevant delivery channel had been surveyed.

Other sources of data, however, support these positive findings. For example, when asked which source of information was most important in influencing their decision to choose MSI services, 31.5% of our sub-Saharan African clients cited a “person who used the service” (Figure 5). Furthermore, 29.9% of our clients in sub-Saharan Africa from across all delivery channels noted that MSI's “good reputation” was the driving force behind their decision to visit an MSI service site (Figure 6). The proportion citing our “good reputation” was also substantial by service delivery channel: 44% of clinic clients, 32% of social franchise clients, and 23% of mobile outreach clients. Such evidence underscores the importance of informal demand generation, based on client acknowledgment of high-quality services and word-of-mouth communication, in influencing MSI client health-seeking behavior for family planning. It also underscores how high-quality service delivery is necessary for expanding access to family planning and scaling up programmatic efforts.

Satisfied clients can help generate demand for family planning services through informal word-of-mouth communication.

## INFRASTRUCTURE AND IMPLEMENTATION STRATEGIES REQUIRED FOR SCALE UP

Underlying MSI's multichannel approach to scaling up delivery of implant services in sub-Saharan Africa were 3 strategies that leveraged and supported key country infrastructure:

1. Provider supply
2. Commodity supply chains
3. Program financing mechanisms

In addition, our experience points to a number of key implementation strategies that should be considered when planning and rolling out programs (Box 3). Finally, operational issues such as access to implant removal services must be planned for in the initial design phase. Each of these factors can pose a barrier to family planning program implementation and expansion if they are not sufficiently addressed.

**BOX 3. Key Implementation Strategies for Scaling Up Delivery of Implants**

**Focus on clients with unmet family planning need.** In order to successfully expand reach, programs must identify and focus on serving prospective users who lack access to a broad range of contraceptive methods, including implants. MSI identifies areas of unmet need through site visits, Ministry of Health input, and analysis of the latest health service and Demographic and Health Survey data.

**Devote resources to raising awareness and diffuse communications through multiple channels.** Sustained awareness-raising activities are critical for attracting new family planning users, including those who choose implants from a wide array of options. Clients may be spread out across a large geographical area and may have limited access to mainstream media channels. Thus, health promotion messages about family planning and implants must be disseminated through different communication channels. Data from MSI client exit interviews in sub-Saharan Africa indicate clients have access to various communication channels, including: community health workers, radio, newspapers, community events, and friends or satisfied clients (Figure 5).

**Deliver high-quality services.** Ensuring high-quality service delivery, at clinical and operational levels, serves as a catalyst for future demand and expansion of service delivery. A positive reputation among clients creates a feedback loop in which existing clients refer new clients. See the Appendix for specific activities MSI uses for establishing service quality.

**Deliver implants through multiple, interconnected service delivery channels.** Using a multipronged strategy to deliver implants helps: (1) ensure the program reaches women of reproductive age in different geographic areas and social strata, as well as with different preferences for health care delivery; (2) generate demand; and (3) ensure comprehensive family planning care for follow up, eventual implant removal, and continued contraceptive use, including family planning counseling and services for clients who do not choose implants. In Madagascar, MSI successfully increased implant uptake and reached the poorest and least accessible women of reproductive age, through its USAID-funded SHOPS (Strengthening Health Outcomes through the Private Sector) program, by using and linking outreach and social franchising channels.^39^

**Build and leverage public-private partnerships.** Given the central role of the Ministry of Health in the health system and its high community visibility, successful private programs work with and strengthen the public health system by: (1) filling gaps in contraceptive method availability, which is sometimes limited to short-acting methods; (2) training public providers in contraceptive counseling and implant removals; and (3) establishing a robust referral system for follow-up care and implant removals. In MSI's SHOPS program in Madagascar, public facilities or providers proved to be the most common referral source for outreach clients and contributed substantially to scaling up implant provision.^39^

### Sufficient Provider Supply

Sufficient health workforce availability and distribution within countries is a key requirement for scaling up implant service delivery.^4^ Unlike condoms or other short-acting methods, implants require a skilled health worker in order for clients to use them. To address health worker deficits, many sub-Saharan African governments have implemented task-shifting and task-sharing initiatives, which increase a country's service delivery capacity by delegating some health care delivery tasks from higher-level to less-specialized health workers.^31^ Various studies have demonstrated the feasibility of these practices for family planning service delivery, and they have proved effective in the scale up of family planning programs, including delivery of implants.^19^^,^^32^^–^^36^ As a result, the World Health Organization (WHO) currently recommends the use of task shifting/sharing for implant delivery, recently endorsing 2 new cadres, auxiliary nurses and lay health workers, for this practice.^37^

WHO recommends task shifting or sharing for implant service delivery to address health worker shortages.

Where allowed by national guidelines, MSI employs task sharing and task shifting to deliver reproductive health and family planning services.^19^^,^^36^ In Ethiopia, Malawi, Mozambique, and Uganda, mid-level providers routinely deliver implants. For example, MSI Ethiopia has dramatically increased its implant delivery capacity through participation in the Integrated Family Health Program, supported by USAID, which has trained more than 10,000 health extension workers to provide implants.

### Strong Supply Chains

Successful health interventions that deliver products to clients in the developing world require robust and predictable commodity supply chains.^38^ Stockouts can reduce service uptake; conversely, a reliable supply of commodities is an important component of high-quality service delivery and can increase uptake and loyalty. MSI's 2012 client exit interview data show that 11.6% of sub-Saharan African clients reported that “services or medicines available” was the most important reason for choosing MSI services (Figure 6).

To ensure a steady supply of implants to its programs in sub-Saharan Africa, MSI uses a **multipronged procurement strategy**. First, MSI country programs work to integrate their supply chains into national supply chains to the greatest extent possible. Large quantities of implants are sourced through Ministry of Health central supplies, many of which are funded by USAID. As funding permits, MSI global headquarters in London also procures implants at bulk prices through international tenders. Implant price-volume guarantees from donors and Implanon and Jadelle manufacturers Merck and Bayer, respectively, allow MSI to secure many more implant units with a finite budget. Additionally, MSI receives a global allocation of implants from the United Nations Population Fund (UNFPA). Together, these international supplies provide the flexibility to smooth out individual countries' implant supplies when shortages occur.

MSI's **product registration initiatives** are another way we strive to ensure availability of implants. MSI works to increase the number of implant brands registered and available in countries. Working in partnership with FHI 360, MSI has registered Sino-implant (II) implants under its branded name Femplant in Burkina Faso, Ghana, and Mali. We have also supported Pharm Access Africa Ltd. in introducing Sino-implant (II) in Kenya, Madagascar, Malawi, Nigeria, Senegal, Sierra Leone, and Tanzania. MSI providers are not limited to using Sino-implant (II) implants, however. They use Implanon and Jadelle brands as well, aiming to meet client preferences regarding the duration of contraceptive protection. However, as MSI typically sources implants through Ministries of Health, the registered brands vary by country, and procurement decisions between brands are often outside of MSI's direct influence. To date, MSI's experience in sub-Saharan Africa shows that demand for implants, and thus program scale up, has occurred regardless of brand.

### Diverse Program Financing Mechanisms

For program scale up in sub-Saharan Africa to be successful, it is essential to reach those underserved clients with the highest unmet need. Unmet need for family planning is higher among low-income sub-Saharan African women than among middle- and higher-income groups.^1^ With 81% of the sub-Saharan African population (in the countries in which MSI works) living on less than US$2.50 per day, the cost of delivering implants must be subsidized to ensure price does not become a barrier to client uptake.^8^ Client exit interview data from 2012 indicate that 9.6% of clients across all service delivery channels in sub-Saharan Africa cited “low-cost” services as the reason why they chose MSI for their family planning services (Figure 6).

MSI uses various financing mechanisms to reduce costs to clients and ensure equity in scale up:

- Part of the surplus generated from clinic operations in developed countries (for example, Australia and the United Kingdom) helps fund the cost of programs in developing countries.
- Any surpluses generated from services for wealthier clients at developing-country clinics help to subsidize services for lower-income clients, primarily mobile outreach services.
- Donor subsidies reduce the true cost of implant service delivery, which encompasses both commodity and operations costs.
- Program efficiencies such as bulk pricing and good logistical management further reduce the cost of service delivery.
- Vouchers distributed in catchment areas with high unmet family planning need and low access to services direct subsidies specifically toward lower-income clients. (MSI uses a needs test to determine eligibility.^40^)

Vouchers enable clients to choose from any participating, accredited provider to receive free family planning services. Over the last 5 years, MSI has piloted and scaled up the use of vouchers in its social franchising networks in certain countries, including Ethiopia, Madagascar, Sierra Leone, and Uganda. In the USAID-funded SHOPS program in Madagascar, the vast majority of social-franchising clients receiving vouchers chose implants. Between January and September 2011, 3,467 LARCs were provided, 3,001 of which were implants (87%). The number of services delivered to non-voucher clients during the same time period remained fairly stable. Thus, the voucher clients did not significantly displace non-voucher clients, indicating market expansion.^39^

In Madagascar, almost 90% of family planning clients receiving vouchers chose implants.

### Implant Removal Services

Contraceptive implants have either a 3-, 4-, or 5-year life span, and clients may decide to discontinue use at any time. Thus, it is essential to have infrastructure in place for implant removals to maintain client trust in the program's family planning services.^7^ Robust and reliable removal services can also help maintain a client as a contraceptive user; removal poses an opportune time to counsel the client on method switching or continuation.

Ensuring reliable implant removal services is essential to maintain client trust in family planning services.

Clients who receive their implants through an MSI clinic or *BlueStar* franchisee typically return to the same location for their removal service or other follow-up care. Outreach clients, however, must be linked to a static site to access removal services or follow-up care when needed. Mobile outreach teams do offer removal services; however, a client may require a removal in the weeks between outreach visits to her catchment area. As part of comprehensive counseling, MSI providers counsel clients on where to go when a removal or follow-up care is required. For clients living far from an MSI clinic or *BlueStar* franchisee, MSI maintains active referral networks of public-sector and, in some cases, other NGO facilities that are trained in implant removal. Clients incur no additional charge for removals as this procedure is considered part of service delivery for implants.

To ensure provider willingness to deliver these removal services, MSI requires that all staff and all social franchise service providers complete competency-based training on implant and IUD removals as well as on management of side effects. Refresher courses occur at regular intervals and are mandatory. Combined with ongoing provider mentoring by MSI's clinical services managers from the country office, these courses aim to bolster provider confidence and knowledge of the procedures for removal and other follow-up care. To date, MSI has not experienced widespread provider reluctance to remove implants, although continued monitoring of this issue is needed.

Maintaining contact with clients after insertion is a key challenge, however. Until recently, MSI, like other family planning service delivery organizations, relied on paper reminder cards to remind clients when to seek implant removals. Since 2012, MSI has been developing a client registration system called the Client Information Center, or CLIC. The system is a combination of software and paper tools that track client profile information including the services and products received during client-provider interactions and any adverse events experienced during the visits.

CLIC has been designed to function in the MSI clinic and at outreach delivery channels, ultimately allowing MSI to track clients between facilities when they present in one location and later in another. Built-in reports allow staff to access information on which clients are due for return visits as well as view user-friendly statistical information on who our clients are and what services they receive over time. If clients wish to share their phone number, it is entered into CLIC so that providers can follow up with appointment reminders, information on minor side effects such as changes in menstruation patterns, information on the timing and location of removal services, and post-removal contraceptive choices. To safeguard confidentiality, clients are contacted by phone only with their permission. Thus, this new system provides MSI with a powerful yet easy-to-use tool to track clients post-procedure, ensuring timely removals of implants at the end of their life span and enabling a better understanding of client follow-up behavior. The use of CLIC may also help mitigate any provider reluctance to perform removals as the electronic record may standardize and normalize removal protocols.

#### Discontinuation and Side Effects

MSI has tracked discontinuation rates and side effects experienced by outreach clients in some sub-Saharan African countries. Only a small proportion of clients surveyed in Ethiopia (0.4%), Sierra Leone (0.7%), and Uganda (2.7%) had discontinued use of implants after 3 months, with rates increasing at later intervals but still remaining low (Table 4).

**TABLE 4.** Implant Discontinuation Rates Among Clients Receiving Implants From MSI in Ethiopia, Sierra Leone, and Uganda, 2010

**Duration of Use**	**Discontinuation Rate**		
	**Ethiopia^a^**	**Sierra Leone^a^**	**Uganda^b^**
	**(N = 562)**	**(N = 433)**	**(N = 470)**
3 months	0.4%	0.7%	2.7%
6 months	0.7%	3.0%	N/A
8 months	5.7%	6.2%	N/A

Abbreviations: MSI, Marie Stopes International; N/A, not applicable.

a Data from Ethiopia and Sierra Leone were collected in April 2010 during retrospective follow-up studies on women who received implants in 2009 at mobile outreach sites.^19^

b Data from Uganda were collected in a prospective cohort study among women receiving implants, IUDs, or tubal ligations between February and April 2010 at mobile outreach sites.^41^

In terms of side effects, only 1.1% of Ugandan clients experienced severe side effects 15 days following insertion; however, none had complications and all received follow-up care.^41^ Severe side effects were defined as a frequent level of discomfort that required medical attention to determine whether a complication had arisen. A much larger proportion, 61.9%, also reported pain around the insertion area at this interval, although these clients did not find it severe. At 6 months post-insertion among clients in Ethiopia and Sierra Leone, the proportion of clients reporting they had ever experienced side effects was 40% and 45%, respectively.^19^ These side effects included cramping and changes in menstrual bleeding that many implant users experience.

**Figure:**
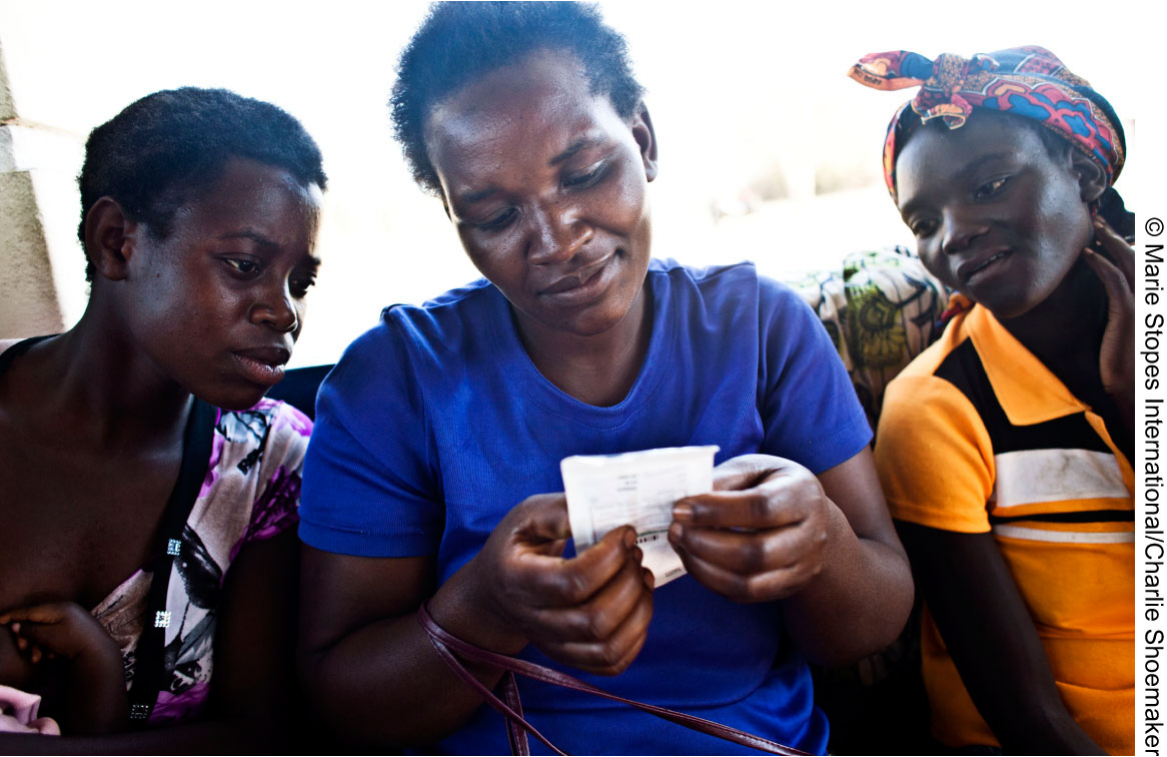
In Zambia, Marie Stopes International clients examine contraceptive implants during a group counseling session about the variety of family planning methods from which women can choose.

## IMPLICATIONS OF MSI'S SERVICE DELIVERY APPROACH

With a cumulative 5-year yield of more than 1.7 million contraceptive implants distributed in sub-Saharan Africa, MSI's family planning service delivery approach can be useful for governments and other organizations aiming for similar program expansion. MSI's experience demonstrates that service delivery expansion can be done successfully in sub-Saharan Africa by leveraging existing service delivery channels that many implementing organizations already use: clinics owned and operated by NGOs, social franchising networks, and mobile outreach teams of dedicated providers that work in partnership with the public sector.

Underlying our channel operations is a strong infrastructure that enables channels to complement each other in user reach and operational structure. Key elements of this infrastructure include a sufficient number of trained providers, strong commodity supply chains, and diverse financing mechanisms. MSI's implementation experience underscores that quality assurance also matters, in the interest of clinical standards but also to help ensure that clients are satisfied with their experience and that they communicate their satisfaction to generate further demand for services.

These systems and strategies have enabled our sub-Saharan African country programs to be nimble in responding to the rising demand for implants over the last 5 years. Governments and organizations wishing to scale up their own programs will likely recognize that the infrastructure investments required to deliver implants as part of a comprehensive method mix can also be leveraged to deliver and expand the uptake of other contraceptive methods.

The adaptive quality of MSI's service delivery models is also an important component of its scale-up efforts in sub-Saharan Africa. In response to changing demand, MSI modified its models to best meet the specific context where family planning service delivery was needed. For example, the mobile community outreach worker team emerged as a low-cost alternative to our original clinical services outreach model, enabling MSI to reach underserved communities in urban and peri-urban areas. A new MSI initiative with the government of Ghana offers another example of a model variation. In this expansion of the public-private partnership component of our outreach model, Ghana Health Services will assume MSI's demand-generation costs for MSI Ghana's mobile outreach channel. Other variations include contracting out opportunities, in which governments contract private-sector implementing organizations to deliver specific services, as MSI has recently established with the government of Tanzania for our outreach services. This adaptation responds to the evolving shift occurring in public-private partnerships, in which governments are assuming greater responsibility for the strategic direction of NGO-provided services (Table 5).

**TABLE 5.** Key Components of Mobile Outreach and Implications for Scale Up, Replication, and Sustainability

**Mobile Outreach Component**	**Implications for:**		
	**Scale Up**	**Replication**	**Sustainability**
Free or highly subsidized services	Helps facilitate rapid expansion, since poor and rural clients have highest unmet need	Requires adequate financing mechanisms to subsidize costs	Requires continued investment and greater role of country governments, through contract arrangements and other innovations
Teams of dedicated providers	Can encourage expansion in areas of high demand by filling service gaps at existing public and private clinics, particularly with high-quality services that can be monitored more easily with such providers	Requires trained staff whocan be deployed to remote areas	Greater emphasis on integrated service delivery models may generate hybrid models. As public-sector capacity develops, dedicated providers may shift their role to a support function.
Public-private partnerships	Must be in place for channel to operate properly, and therefore, for service delivery expansion to occur	Requires collaborative relationships with public sector and robust referral systems	Possible to sustain over the long term, although dynamics may change with the private sector mentoring public-sector providers who assume a larger role in service delivery (presuming the supply of competent public providers increases)

Looking forward, the increasing availability of implants will generate demand, and growing numbers of women in sub-Saharan Africa are likely to choose this method. Our recent results in the region, in which every country where we work produced steep rates of growth, demonstrate this demand; our data also show that implant service delivery, among other contraceptive methods, still has room to expand. Concurrent with this rising demand for implant insertion services will be an increase in the need to remove implants. As early users reach the end of their implant's life span, clients will seek removals in greater numbers than before. Such demand for removals will need to be met with additional family planning services in the context of informed choice; post-removal contraceptive counseling services and method choice availability are key for women who wish to continue using a contraceptive method following the removal.

Meeting sustained demand for implant insertion, removal, and post-removal services in the long term will require MSI and other service delivery organizations to develop innovative responses to changing needs and to forge strategic partnerships between stakeholders, including clients. The public-private partnerships that have brought us to the current stage in implant scale up—including the price-volume guarantees and the partnerships between NGOs and local governments that underpin outreach and dedicated provider models—set the tone for further collaboration. Rather than viewing mobile outreach, dedicated provider, and social franchising models as stop-gap measures to support shortfalls in public- or private- (commercial) sector capacity, organizations may be able to integrate these models into the existing health system. MSI's new contract models with the governments of Ghana and Tanzania are examples of this integration. Other sustainability strategies include the incorporation of social franchise clinics in national and social health insurance schemes, and publicly funded voucher programs delivering free or very low-cost services for the poorest clients.

As donors, governments, and implementing partners work to reach 120 million additional contraceptive users by 2015 as part of the Family Planning 2020 (FP2020) goals, responsiveness within the global health community will be essential. With the recent price-volume guarantees on implants from manufacturers and donors, important progress has already been made in reducing the financial burden of implant procurement. However, continued investment in the implementation costs required for reaching the client is essential—as a “service-volume guarantee” to meet demand among all current and future clients. Taken together, such investments in commodity supplies and effective, high-quality service delivery will enable all of us to deliver on our FP2020 commitments, and ultimately, ensure that all individuals have access to their contraceptive method of choice.

## Footnotes

* MSI's social franchise networks are branded *BlueStar* with the exception of *Plan.It* (India), *Rayaheen* (Yemen), *Suraj* (Pakistan), and *Amua* (Kenya). In this article, *BlueStar* is used to refer to all MSI franchise networks in sub-Saharan Africa, including *Amua* in Kenya.

## Appendix. Quality Assurance Measures and Monitored Service Delivery Components by Marie Stopes International (MSI)

### Quality Assurance Measures

**Clinical protocols and franchise operational procedures**

- All MSI providers in all channels adhere to MSI's Clinical Standards and Guidelines as well as their country's Ministry of Health protocols
- Comprehensive follow-up care required, including referrals to MSI or public-sector facilities for complications or implant removal
- All social franchisees are trained in operational, marketing, and quality-control protocols, such as record keeping and commodity management
- Interactive, competency-based, and up-to-date trainings and refresher courses

**Regular supervision**

- Supportive supervision to all provider and operations teams in all channels, including social franchising, by MSI country program offices, regionally-based clinical staff, and London headquarters staff
- Some social franchisees engage providers in regular network meetings
- Programs operate under measurable quarterly and annual goals established under MSI's monitoring and evaluation framework

**Intermittent quality checks**

- Random spot checks from MSI clinical services managers, typically each quarter
- Mystery client visits for family planning services, including implants
- Anonymous complaint and support telephone lines for MSI clinic and social franchisee clients
- Comprehensive Quality Technical Assistance audits by MSI London's Medical Development Team to review clinical quality, on an annual basis

### Service Delivery Areas Monitored

**Clinical quality of implant service provision**

- Client family planning counseling
- Implant insertion
- Infection prevention
- Management of follow-up care, including referral systems

**Service delivery environment**

- Customer service
- Facility cleanliness
- Privacy

**Program operations**

- Personnel management
- Commodities management
- Record keeping
- Demand generation
- Collaboration with local Ministry of Health facilities and staff, when applicable

## References

[1] Singh S Darroch JE Adding it up: costs and benefits of contraceptive services—estimates for 2012. New York: Guttmacher Institute; 2012 Available from: http://www.guttmacher.org/pubs/AIU-2012-estimates.pdf

[2] United Nations Department of Economic and Social Affairs. Population Division. World contraceptive use 2011. New York: United Nations; 2012 Available from: http://www.un.org/esa/population/publications/contraceptive2011/contraceptive2011.htm

[3] Westoff CF New estimates of unmet need and the demand for family planning. DHS Comparative Reports No. 14. Calverton (MD): Macro International Inc; 2006 Available from: http://www.measuredhs.com/pubs/pdf/CR14/CR14.pdf

[4] Wickstrom J Jacobstein R Contraceptive security: incomplete without long-acting and permanent methods of family planning. Stud Fam Plann. 2011;42(4): 291–298 10.1111/j.1728-4465.2011.00292.x 22292248

[5] Van Lith LM Yahner M Bakamjian L Women's growing desire to limit births in sub-Saharan Africa: meeting the challenge. Glob Health Sci Pract. 2013;1(1): 97–107 10.9745/GHSP-D-12-00036 PMC4168554 25276520

[6] Cleland JG Ndugwa RP Zulu EM Family planning in sub-Saharan Africa: progress or stagnation? Bull World Health Organ. 2011;89(2): 137–143 10.2471/BLT.10.077925 21346925 PMC3040375

[7] Jacobstein R Stanley H Contraceptive implants: providing better choice to meet growing family planning demand. Glob Health Sci Pract. 2013;1(1): 11–17 10.9745/GHSP-D-12-00003 PMC4168562 25276512

[8] Hayes G Fry K Weinberger M Global impact report 2012: reaching the under-served. London: Marie Stopes International; 2013 Available from: http://www.mariestopes.org/sites/default/files/Global-Impact-Report-2012-Reaching-the-Under-served.pdf

[9] Bayer HealthCare Press Center. Bayer joins global initiative for better access to safe and effective contraception [press release]. [Leverkusen (Germany)]: Bayer HealthCare; 2012 9 26 [cited 2013 Aug 7]. Available from: http://press.healthcare.bayer.com/en/press/news-details-page.php/14732/2012-0429

[10] Merck (MSD). MSD and partners announce agreement to increase access to innovative contraceptive implants Implanon® and Implanon NXT® in the poorest countries. Whitehouse Station (NJ): MSD; 2013 [cited 2013 Aug 7]. Available from: http://www.rhsupplies.org/fileadmin/user_upload/Announcements/MERCK_EXTERNAL_STATEMENT_FINAL_May_2013__4_.pdf

[11] Department for International Development (DFID). Choices for women: planned pregnancies, safe births and healthy newborns. The UK's Framework for Results for improving reproductive, maternal and newborn health in the developing world. London; DFID; 2010 Available from: https://www.gov.uk/government/uploads/system/uploads/attachment_data/file/67640/RMNH-framework-for-results.pdf

[12] Family Planning Summit Metrics Group. Technical note: data sources and methodology for calculating the 2012 baseline, 2020 objectives, impacts and costings. [place unknown]: Family Planning Summit; 2012

[13] United Nations Population Fund (UNFPA). Programme of action adopted at the International Conference on Population and Development, Cairo, 5–13 September 1994. New York: UNFPA; 1995 12157972

[14] U.S. Agency for International Development (USAID). USAID's family planning guiding principles and U.S. legislative and policy requirements. Standard provisions for nongovernmental organizations: a mandatory reference for ADS Chapter 303. Washington, DC: USAID; 2012

[15] RamaRao S Mohanam R The quality of family planning programs: concepts, measurements, interventions, and effects. Stud Fam Plann. 2003;34(4): 227–248 10.1111/j.1728-4465.2003.00227.x 14758606

[16] Cleland J Bernstein S Ezeh A Faundes A Glasier A Innis J Family planning: the unfinished agenda. Lancet. 2006;368(9549): 1810–1827 10.1016/S0140-6736(06)69480-4 17113431

[17] Marie Stopes International (MSI), Research, Monitoring & Evaluation Team, Evidence & Innovation, Health System Department. M&E manual, version 2: strengthening M&E across the MSI partnership. London: MSI; 2013

[18] Neukom J Chilambwe J Mkandawire J Mbewe RK Hubacher D Dedicated providers of long-acting reversible contraception: new approach in Zambia. Contraception. 2011;83(5): 447–452 10.1016/j.contraception.2010.08.021 21477688

[19] Eva G Ngo TD MSI mobile outreach services: retrospective evaluations from Ethiopia, Myanmar, Pakistan, Sierra Leone and Viet Nam. London: Marie Stopes International; 2010 Available from: http://www.mariestopes.org/sites/default/files/outreach_web.pdf

[20] Tchedre A Naugle D Mobile outreach dismantles barriers to the adoption of long term family planning methods in Togo. Presented at: 138th Annual Meeting of the American Public Health Association; 2010 Nov 6–10; Denver, CO. Abstract available from: https://apha.confex.com/apha/138am/webprogram/Paper223803.html

[21] Reichwein B Weinberger M Fry K Nuccio O Meeting FP2020 commitments: the importance of moving beyond first time users. MSI Research Brief Series 2013/004. London: Marie Stopes International; 2013 Available from: http://www.mariestopes.org/sites/default/files/Reaching%20FP2020%20goals_The%20importance%20of%20moving%20beyond%20first%20time%20users.pdf

[22] Kifle YA Nigatu TH Cost-effectiveness analysis of clinical specialist outreach as compared to referral system in Ethiopia: an economic evaluation. Cost Eff Resour Alloc. 2010;8(1): 13 10.1186/1478-7547-8-13 20540766 PMC2892431

[23] Campbell J Corby N Increasing family planning access and choice: key lessons from Marie Stopes International's clinical outreach programmes. London: Marie Stopes International; 2011 Available from: http://www.mariestopes.org/sites/default/files/12pp_Marie%20Stopes_Marketing_Outreach%20WEB.pdf

[24] Ngo AD Alden DL Pham V Phan H The impact of social franchising on the use of reproductive health and family planning services at public commune health stations in Vietnam. BMC Health Serv Res. 2010;10:54 10.1186/1472-6963-10-54 20187974 PMC2845125

[25] Huntington D Mundy G Hom NM Li Q Aung T Physicians in private practice: reasons for being a social franchise member. Health Res Policy Syst. 2012;10(1): 25 10.1186/1478-4505-10-25 22849434 PMC3464134

[26] Stephenson R Tsui AO Sulzbach S Bardsley P Bekele G Giday T Franchising reproductive health services. Health Serv Res. 2004;39(6 Pt 2): 2053–2080 10.1111/j.1475-6773.2004.00332.x 15544644 PMC1361112

[27] Bruce J Fundamental elements of the quality of care: a simple framework. Stud Fam Plann. 1990;21(2): 61–91 10.2307/1966669 2191476

[28] Hutchinson PL Do M Agha S Measuring client satisfaction and the quality of family planning services: a comparative analysis of public and private health facilities in Tanzania, Kenya and Ghana. BMC Health Serv Res. 2011;11:203 10.1186/1472-6963-11-203 21864335 PMC3224259

[29] Williams T Schutt-Ainé J Cuca Y Measuring family planning service quality through client satisfaction exit interviews. Int Fam Plan Perspect. 2000;26(2): 63–71 10.2307/2648269

[30] Blanc AK Curtis SL Croft TN Monitoring contraceptive continuation: links to fertility outcomes and quality of care. Stud Fam Plann. 2002;33(2): 127–140 10.1111/j.1728-4465.2002.00127.x 12132634

[31] World Health Organization (WHO). Task shifting to tackle health worker shortages. Geneva: WHO; 2007 Available from: http://www.who.int/healthsystems/task_shifting_booklet.pdf

[32] Janowitz B Stanback J Boyer B Task sharing in family planning. Stud Fam Plann. 2012;43(1): 57–62 10.1111/j.1728-4465.2012.00302.x 23185872

[33] Hoke TH Wheeler SB Lynd K Green MS Razafindravon BH Rasamihajamanana E Community-based provision of injectable contraceptives in Madagascar: ‘task shifting' to expand access to injectable contraceptives. Health Policy Plan. 2012;27(1): 52–59 10.1093/heapol/czr003 21257652

[34] Malarcher S Meirik O Lebetkin E Shah I Spieler J Stanback J Provision of DMPA by community health workers: what the evidence shows. Contraception. 2011;83(6): 495–503 10.1016/j.contraception.2010.08.013 21570545

[35] World Health Organization (WHO). From evidence to policy: expanding access to family planning. Optimizing the health workforce for effective family planning services. Geneva: WHO; 2012 Available from: http://apps.who.int/iris/bitstream/10665/75164/1/WHO_RHR_HRP_12.19_eng.pdf

[36] Pernito V Nelson E Wallach S Scaling up the delivery of rural services in the Philippines. London: Marie Stopes International; 2009 Available from: http://www.mariestopes.org/sites/default/files/Scaling_up_delivery_of_rural_outreach_in_the_Philippines.pdf

[37] World Health Organization (WHO). WHO recommendations: optimizing health worker roles to improve access to key maternal and newborn health interventions through task shifting. Geneva: WHO; 2012 Available from: http://apps.who.int/iris/bitstream/10665/77764/1/9789241504843_eng.pdf 23844452

[38] John Snow, Inc (JSI). Getting products to people: the JSI framework for integrated supply chain management in public health. Arlington (VA): JSI; 2012 Available from: http://www.jsi.com/JSIInternet/Inc/Common/_download_pub.cfm?id=11907&lid=3

[39] Kemplay M Neggaz M Mani N Madagascar program profile. Bethesda (MD): Abt Associates, Strengthening Health Outcomes through the Private Sector Project; 2013 Available from: http://shopsproject.org/sites/default/files/resources/Madagascar%20Program%20Profile.pdf

[40] Boler T Harris L Reproductive health vouchers: from promise to practice. London: Marie Stopes International; 2010 Available from: http://www.mariestopes.org/sites/default/files/vouchers-from-promise-to-practice.pdf

[41] Reiss K Nantayi L Odong J Ngo TD Providing long-acting and permanent contraceptives through outreach in rural Uganda. London: Marie Stopes International; 2012 Available from: http://www.mariestopes.org/sites/default/files/MSU%20Outreach%20Evaluation%20August%202012%20WEB.pdf

